# Association of hemoglobin levels with bone mineral density for adults over 18 years of age: a cross-sectional study

**DOI:** 10.1038/s41598-022-13973-w

**Published:** 2022-06-15

**Authors:** Enqi Liu, Xinzheng Hou, Siqi Liu, Jing Han, Hao Lv

**Affiliations:** 1 College of Acupuncture and Massage, Shandong University of Traditional Chinese Medicine, Shandong, 250355 China; 2 College of Traditional Chinese Medicine, Shandong University of Traditional Chinese Medicine, Shandong, 250355 China; 3 Acupuncture and Massage Rehabilitation Department, Qingdao Traditional Chinese Medicine Hospital, 4 Renmin Road, Qingdao City, 266033 Shandong Province China; 4 Department of Acupuncture, Affiliated Hospital of Shandong University of Traditional Chinese Medicine, 16369 Jingshi Road, Jinan City, 250014 Shandong Province China; 5 Pediatric Orthopaedics, Affiliated Hospital of Shandong University of Traditional Chinese Medicine, 16369 Jingshi Road, Jinan City, 250014 Shandong Province China

**Keywords:** Medical research, Outcomes research

## Abstract

The overall objective of this study was to determine the association between hemoglobin (HGB) and bone mineral density (BMD) in the lumbar and thoracic spine of adults aged ≥ 18 years. This cross-sectional study utilized the non-institutionalized US population from the National Health and Nutrition Survey (NHANES) as the sample source. A multiple linear regression model was used to assess the relationship between HGB and BMD in the lumbar and thoracic spine, with analysis of subgroups conducted according to sex and race. Smooth curve fitting was performed to explore the potential nonlinear relationship. When nonlinearity was found, we further constructed a weighted two-piecewise linear regression model and used a recursive algorithm to calculate the inflection point. After accounting for relevant confounding variables, HGB was found to be negatively associated with lumbar spine BMD in multiple regression models. However, in the subgroup analyses stratified by sex and race, the relationship between HGB and thoracic spine BMD and lumbar spine BMD was only found in women and other races and races that were not recorded. In Non-Hispanic Asian subjects, the relationship between HGB and BMD in the lumbar spine and thoracic spine showed a U-shaped curve. In addition, the relationship between HGB and BMD in the lumbar spine formed an inverted U-shaped curve among participants in other races and those whose race was not reported. Our study shows that HGB has a non-linear relationship with lumbar and thoracic BMD. Further studies are required to elucidate the mechanisms underlying this association.

## Introduction

Osteoporosis, which most commonly occurs in the hip, spine, and wrist, not only puts people at an increased risk of fragility and fracture but also has a substantial impact on their families and the society^1,2^. Bone mineral density (BMD) evaluated using the Dual energy X-ray absorptiometry (DXA)^3^, accurately captures bone mass and is the gold standard for diagnosing osteoporosis, as well as being the best predictor of osteoporotic fracture risk^4^. BMD is affected by several factors, including sex, age, genetic factors, nutrition, hormone levels, physical activity, smoking, and alcohol consumption^5^. A recent study by Korkmaz et al. showed that anemia was an independent risk factor for low BMD in postmenopausal women^6^.

Hemoglobin (HGB) is found in erythrocytes, and its role is to combine with oxygen and transport it from the lungs to tissues^7^. Anemia, characterized by 'low hemoglobin concentration', is a major public health problem prevalent in low-, middle-, and high-income countries worldwide^8^, affecting nearly a quarter of the world's population and predominantly affecting women and pre-school children^9^. Low levels of HGB are recognized as risk factors for osteoporosis, falls, fractures, and physical decline in the elderly population^10,11^.

Zhou et al. conducted a study on 1238 volunteers from Anhui, China, and obtained a U-shaped curve for the relationship between BMD and HGB in healthy men^12^. In contrast, a positive correlation was found between HGB levels and BMD in patients with chronic obstructive pulmonary disease, chronic kidney disease, sickle cell anemia, and inflammatory bowel disease^13–17^. In addition, Zarei et al. measured BMD and bone mineral content in the lumbar spine and femoral neck of 21 patients over 10 years of age with hemoglobin H disease and concluded that the prevalence of BMD in the lumbar spine and femoral neck region in patients with hemoglobin H disease was significantly lower than the expected age range compared to healthy individuals^18^. Cui et al. adjusted for age, duration of diabetes, body mass index, and alanine aminotransferase to conclude that anemia was associated with osteoporosis in patients with type 2 diabetes mellitus, irrespective of sex^19^. Research on the relationship between HGB and BMD has been limited by the selection of study populations and sample sizes, and the findings are inconsistent. Furthermore, only a few studies have thoroughly investigated the association between HGB and thoracic and lumbar BMD.

Therefore, this cross-sectional study was conducted using the 2013–2018 National Health and Nutrition Survey (NHANES) as a sample source to determine the association between HGB and BMD in the lumbar and thoracic spine in adults aged ≥ 18 years.

## Materials and methods

### Study population

Data for the analysis were obtained from the NHANES (2013–2018), a large, comprehensive, and regularly updated probability sample of the non-institutionalized U.S. population^20–22^.

The study population consisted of participants aged ≥ 18 years who had complete data on HGB and lumbar and thoracic spine BMD. The NCHS Ethics Review Committee approved the implementation of the NHANES, and informed consent was obtained from all participants. The study adhered to the relevant guidelines and regulations. The encryption procedure was uniform to make it possible to link complaints belonging to the same patient in the NHANES. Detailed documentation of the ethics application and written informed consent are available on the NHANES website^23–25^.

### Data collection

The following information was collected by two researchers (XZH and SQL):

Demographic data [gender, age, race, annual household income and education level].

Examination data [BMD of the lumbar spine and the thoracic spine (mg/cm^2^), systolic blood pressure (SBP) (mmHg), diastolic blood pressure (DBP) (mmHg), body mass index (BMI) (kg/m^2^)].

Laboratory data [HGB (g/dL), red blood cell count (× 10^6^ cells/μL), albumin creatinine ratio (mg/g), albumin (g/L), alkaline phosphatase (ALP)(IU/L), alanine aminotransferase (ALT)(U/L), blood urea nitrogen (mmol/L), total calcium (mg/dL), iron (umol/L), creatinine phosphor kinase (CPK)(IU/L), glucose (mmol/L), lactate dehydrogenase (LDH)(IU/L), phosphorus (mmol/L), potassium (mmol/L), total bilirubin (umol/L), total protein (g/L), creatinine (umol/L), uric acid (umol/L), blood cadmium (μg/L),blood lead (μg/L),triglyceride (TG) (mmol/L),low-density lipoprotein cholesterol (LDL-C)(mmol/L), high-density lipoprotein cholesterol (HDL-C)(mmol/L) and cholesterol level (mmol/L)].

Questionnaire data [alcohol status (average alcohol drinks/day in the past 12 months), smoking status, diabetes (Has a doctor told you that you have diabetes?), rheumatoid arthritis (RA) (Which type of arthritis was it?), chronic obstructive pulmonary disease (COPD) (Ever told you had copd?), cancer or malignancy (Ever told you had cancer or malignancy?)].

Weight value [Depending on the rules for selecting weight values offered on the NHANES website, “Full Sample Two-Year Mobile Examination Center Exam Weight (WTMEC2YR)” was Selected as representative weighting value].

For the selection of confounding variables, we followed the following principles: (1) based on clinical significance, we selected indicators related to HGB and BMD; (2) from the perspective of statistical methodology, we chose to include factors that have an impact greater than 10% on the results; (3) based on the above two points, confounding factors were selected according to the biological point of view.

Treatment of missing values for variables: (1) the missing values of continuous variables were replaced with mean values; (2) the missing values of a categorical variable were separated into a "not recorded" group.

### Evaluation of variables

HGB was measured at the NHANES Mobile Examination Centers (MECs). A Beckman Coulter DxH 800 instrument was used to obtain a complete blood count (CBC) on blood specimens and provide a distribution of blood cells for all participants. Transmittance of light at 525 nm through a lysed WBC solution in the HGB cuvette was compared to the transmittance of the same light through a reagent blank. The system converted this ratio to an HGB value using a calibration factor. The weight (mass) of HGB was determined by the degree of absorbance recorded from the photocurrent transmittance expressed in g/dl. A detailed description of the measurement of HGB is mentioned in the Laboratory Method Files section on the NHANES website^26^.

The low-level X-rays of the Hologic Discovery model A densitometer (Hologic, Inc., Bedford, Massachusetts) with Apex 3.2 software was used to scan the patient's body to estimate BMD under standard operating conditions by radiographers who participated in the DXA examination; the entrance dose of the examinee for a whole-body scan was less than 1 mR^1^ (a standard X-ray is approximately 35 R). More information regarding the measurement of the DXA examination protocol can be obtained from the NHANES website^27^.

Information about age, gender, race, education level, annual household income, alcohol status, smoking status, diabetes, RA, and COPD was provided to the participants by trained interviewers using a computer-assisted personal interview (CAPI) system. Data for serum albumin, blood urea nitrogen, uric acid, phosphorus, ALP, ALT, iron, CPK, glucose, LDH, potassium, total bilirubin, total protein, creatinine and calcium were obtained from standard biochemical profile analysis using a Beckman Synchron LX20. Total cholesterol were analyzed using a Roche Modular P chemistry analyzer (enzymatic method). Metals determination for blood cadmium and blood lead was performed by inductively coupled plasma mass spectrometry on whole blood samples from the Laboratory Sciences Department of the National Center for Environmental Health. Triglycerides (TG), low density lipoprotein cholesterol (LDL-C) and high density lipoprotein cholesterol (HDL-C).The details of the variables mentioned above are available on the NHANES website^28^ and Appendix 1.

### Statistical analysis

We used the weighted chi-square test to calculate the differences between the classification variables and a weighted linear regression model for continuous variables. Finally, the means (continuous variables) or proportions (categorical variables) were used to describe the baseline characteristics of all participants included in the study. Weighted multiple linear regression analysis was used to calculate the relationship between HGB and BMD. Following the Strengthening the Reporting of Observational Studies in Epidemiology (STROBE) guidelines^29^, we calculated the unadjusted, minimally adjusted, and fully adjusted results. To further investigate the relationship between HGB and BMD of the thoracic and lumbar spine in different populations, a subgroup analysis was performed by separating the participants by age and sex. Additionally, smooth curve fitting was performed to explore the nonlinear relationship. Accordingly, we further constructed a weighted two-piecewise linear regression model and used a recursive algorithm to calculate the inflection point.

P values < 0.05 were defined as significant. All analyses were undertaken by EmpowerStats (version: 2.0. X&Y Solutions, Inc, Boston, MA. http://www.empowerstats.com) and R software, v.3.4.3 (Vienna, Austria: R Foundation for Statistical Computing, 2016 http://www.R-project.org).

### Ethics approval

All analyses were based on data of the National Health and Nutrition Examination Survey (NHANES). And all procedures performed in studies involving human participants were in accordance with the ethical standards of the institutional and/or national research committee and with the 1964 Helsinki declaration and its later amendments or comparable ethical standards. The study was approved by the ethics review board of the National Center for Health Statistics. The National Center for Health Statistics Ethics Review Board protocol numbers are Protocol #2011-17 (NHANES 2013–2014), Continuation of Protocol #2011-17 (NHANES 2015–2016), Continuation of Protocol #2011-17 and #2018-01 (NHANES 2017–2018), respectively. The detailed information located on the NHANES website.

### Consent for publication

All participating authors give their consent for this work to be published.

## Results

### Participant selection and general characteristics

**Figure 1:**
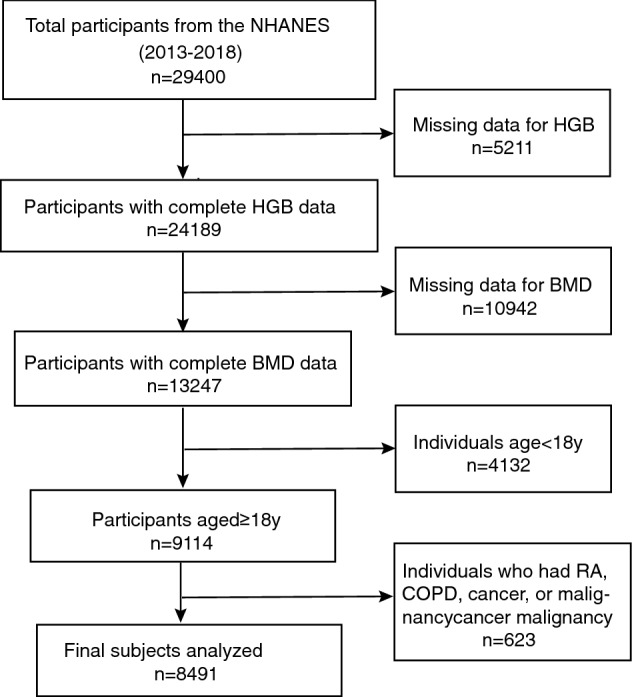
Flow chart of participants selection.

Initially, the information of 29,400 participants was extracted from the NHANES database for 2013–2018. After excluding individuals without HGB data (n = 5211) and BMD data (n = 10,942), under 18 years of age (n = 4133), and with RA, COPD, cancer, or any other malignancy (n = 623), a total of 8491 participants were included in this study (Fig. 1).

The weighted socio-demographic and medical characteristics of the participants are presented in Table 1. The mean age of these participants was 37.8 ± 12.1 years, of which 52.5% were male and 47.2% were female, with non-Hispanic whites being the most numerous. There were significant differences in sex, age, race, annual household income, education level, SBP, DBP, BMI, red blood cell count, albumin, ALP, ALT, blood urea nitrogen, total calcium, iron, CPK, creatinine, glucose, LDH, phosphorus, total bilirubin, total protein, uric acid, albumin creatinine ratio, blood cadmium, blood lead, phosphorus, potassium, triglyceride, LDL-C, HDL-C, total cholesterol, alcohol status, smoking status and diabetes across the different HGB groups (quartiles, Q1 to Q4).

**Table 1:** Weighted characteristics of the study population.

Hemoglobin (g/dL)	All	Q1	Q2	Q3	Q4	P-value
Age	37.8 ± 12.1	37.9 ± 11.6	38.3 ± 12.2	38.3 ± 12.2	36.9 ± 12.1	< 0.0001
**Sex (%)**						< 0.0001
Male	52.5	6.3	24.3	66.2	94.7	
Female	47.5	93.7	75.7	33.8	5.3	
**Race (%)**						< 0.0001
Mexican American	15.0	14.4	15.7	14.2	15.6	
Other Hispanic	9.7	10.8	9.6	10.4	8.6	
Non-Hispanic White	42.4	38.0	41.8	43.9	44.6	
Non-Hispanic Black	18.1	22.0	18.1	17.6	15.8	
Non-Hispanic Asian	9.4	9.9	9.7	8.3	9.9	
Other Race and not recorded	5.3	5.0	5.2	5.6	5.5	
**Education level (%)**						< 0.0001
Less than 9th grade	3.9	3.7	3.3	3.8	4.5	
9–11th grade (Includes 12th grade with no diploma)	8.3	8.2	7.6	7.8	9.4	
High school graduate/GED or equivalent	21.6	17.7	20.6	23.4	23.4	
Some college or AA degree	30.8	31.8	30.3	31.5	30.0	
College graduate or above	30.7	33.1	34.1	29.7	27.3	
Not recorded	4.7	5.4	4.1	3.8	5.4	
**Annual household income**						0.0093
$ 0–4999	1.7	2.3	1.2	1.5	1.7	
$ 5000–9999	2.0	2.4	1.7	2.3	1.6	
$10,000–14,999	2.8	3.2	2.8	2.2	2.8	
$15,000–19,999	3.4	4.4	3.2	3.3	3.1	
$20,000–24,999	4.6	5.1	4.0	4.1	5.0	
$25,000–34,999	8.0	8.2	7.3	8.5	8.1	
$35,000–44,999	8.8	8.7	7.6	8.4	10.1	
$45,000–54,999	8.0	8.6	8.6	6.6	8.3	
$55,000–64,999	6.4	5.9	6.2	6.3	6.8	
$65,000–74,999	5.4	5.2	5.2	6.1	5.0	
$20,000 and over	3.2	3.0	3.0	3.1	3.7	
Under $20,000	0.8	0.8	1.1	0.8	0.4	
$75,000–99,999	12.4	11.4	12.2	12.5	13.3	
$100,000 and over	27.8	25.6	31.3	29.6	24.8	
Not recorded	4.9	5.2	4.4	4.6	5.3	
**Alcohol status (%)**						< 0.0001
< 12 oz	73.0	68.0	72.0	75.3	75.2	
≥ 12 oz	1.6	0.3	0.7	1.7	3.2	
Not recorded	25.4	31.7	27.3	23.0	21.6	
**Smoking status (%)**						< 0.0001
Every day	14.9	10.3	13.9	18.9	22.8	
Some days	4.7	3.5	3.8	4.1	6.8	
Not at all	18.2	11.2	15.9	19.0	18.7	
Not recorded	62.2	75.0	66.3	58.1	51.7	
**Diabetes (%)**						0.0163
Yes	5.1	6.2	4.7	4.8	5.0	
No	93.1	91.6	93.6	93.8	93.2	
Borderline	1.7	2.1	1.6	1.5	1.8	
Not recorded	0.0	0	0.2	0	0	
BMI (kg/m^2^)	28.9 ± 6.9	29.2 ± 7.9	28.9 ± 7.3	28.7 ± 6.5	29.1 ± 6.2	0.1095
SBP (mmHg)	118.5 ± 13.8	116.7 ± 14.3	116.8 ± 14.2	119.0 ± 13.5	120.9 ± 12.9	< 0.0001
DBP (mmHg)	71.2 ± 11.1	69.2 ± 10.7	70.3 ± 11.0	71.5 ± 11.1	73.0 ± 11.2	< 0.0001
Thoracic spine BMD	8.2 ± 1.2	8.1 ± 1.1	8.1 ± 1.1	8.3 ± 1.1	8.4 ± 1.1	< 0.0001
Lumbar spine BMD	10.3 ± 1.5	10.6 ± 1.4	10.5 ± 1.5	10.3 ± 1.5	10.3 ± 1.5	< 0.0001
Albumin (g/L)	43.2 ± 3.4	41.1 ± 3.3	42.7 ± 3.0	43.6 ± 3.1	44.6 ± 3.2	< 0.0001
ALP (IU/L)	68.1 ± 23.5	65.5 ± 22.7	66.5 ± 22.1	68.0 ± 24.0	71.2 ± 24.4	< 0.0001
ALT (IU/L)	26.0 ± 19.9	19.4 ± 13.4	22.2 ± 16.7	26.0 ± 17.0	33.6 ± 25.2	< 0.0001
Blood urea nitrogen (mmol/L)	4.7 ± 1.5	4.3 ± 1.7	4.5 ± 1.3	4.8 ± 1.5	4.8 ± 1.4	< 0.0001
Total calcium (mmol/L)	2.3 ± 0.1	2.3 ± 0.1	2.3 ± 0.1	2.4 ± 0.1	2.4 ± 0.1	< 0.0001
CPK (IU/L)	167.5 ± 267.6	128.8 ± 158.5	147.2 ± 235.3	187.8 ± 315.3	192.2 ± 298.9	< 0.0001
Creatinine (umol/L)	75.6 ± 24.2	67.9 ± 41.6	69.8 ± 16.4	77.9 ± 16.0	83.5 ± 14.6	< 0.0001
LDH (IU/L)	132.0 ± 29.7	128.4 ± 28.4	131.1 ± 29.5	133.0 ± 29.0	134.2 ± 31.0	< 0.0001
Phosphorus (mmol/L)	1.2 ± 0.2	1.2 ± 0.2	1.2 ± 0.2	1.2 ± 0.2	1.2 ± 0.2	1.2 ± 0.2
Potassium (mmol/L)	4.0 ± 0.3	3.9 ± 0.3	4.0 ± 0.3	4.0 ± 0.3	4.0 ± 0.3	< 0.0001
Total bilirubin (umol/L)	9.9 ± 5.3	7.8 ± 4.1	9.1 ± 4.7	9.9 ± 4.9	11.8 ± 6.2	7.8 ± 4.1
Total protein (g/L)	71.5 ± 4.2	70.8 ± 4.4	71.2 ± 4.0	71.7 ± 4.1	72.2 ± 4.1	< 0.0001
Albumin creatinine ratio (mg/g)	24.3 ± 234.5	47.1 ± 394.2	27.2 ± 289.9	11.5 ± 52.2	17.7 ± 94.8	< 0.0001
Red blood cell count (× 10^6^ cells/μL)	4.8 ± 0.5	4.4 ± 0.4	4.6 ± 0.3	4.9 ± 0.3	5.2 ± 0.3	< 0.0001
HDL-C (mmol/L)	1.4 ± 0.4	1.5 ± 0.4	1.5 ± 0.4	1.4 ± 0.4	1.2 ± 0.3	< 0.0001
Total cholesterol (mmol/L)	4.9 ± 1.0	4.7 ± 0.9	4.9 ± 1.0	4.9 ± 1.0	5.0 ± 1.1	< 0.0001
Glucose (mmol/L)	5.4 ± 1.8	5.3 ± 1.5	5.3 ± 1.7	5.4 ± 1.8	5.5 ± 2.0	< 0.0001
Iron (umol/L)	15.9 ± 6.8	12.2 ± 6.6	15.1 ± 5.8	16.7 ± 6.5	18.4 ± 6.6	< 0.0001
Uric acid (umol/L)	319.1 ± 80.8	272.9 ± 68.9	292.1 ± 70.5	333.0 ± 79.2	359.9 ± 73.1	< 0.0001
Blood cadmium (μg/L)	0.4 ± 0.4	0.4 ± 0.3	0.4 ± 0.3	0.4 ± 0.4	0.4 ± 0.4	0.0078
Blood lead (μg/L)	1.0 ± 1.1	0.9 ± 0.7	0.9 ± 0.7	1.0 ± 1.0	1.2 ± 1.6	< 0.0001
Triglyceride (mmol/L)	1.7 ± 1.4	1.4 ± 0.9	1.5 ± 1.1	1.6 ± 1.1	2.1 ± 1.8	< 0.0001
**LDL-C (mmol/L) (%)**						< 0.0001
0–2.302	11.3	11.4	11.5	12.3	10.0	
2.301–2.870	13.1	11.7	13.3	12.5	14.3	
2.871–3.491	11.0	10.4	9.6	11.7	12.1	
> 3.491	10.3	7.4	7.9	11.0	13.6	
Not recorded	54.3	59.1	57.6	52.6	49.9	

Mean ± SD for continuous variables: P-value was calculated by weighted linear regression model.

% for categorical variables: P-value was calculated by weighted chi-square test.

### Relationship between HGB and BMD

To explore the relationship between HGB and BMD, we constructed three weighted univariate and multivariate linear regression models: Model 1, unadjusted; Model 2, adjusted for sex, age, and race; and Model 3, adjusted for the covariates in Table 1.

**Figure 2:**
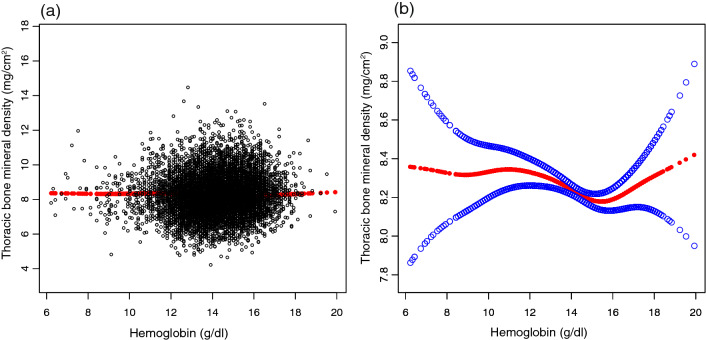
Correlation between hemoglobin and thoracic bone mineral density. (**a**) Each black point represents one sample. (**b**) The area between the two blue dashed lines is represented as a 95% CI. The area between the lines is expressed as 95% CI. Each point represents the size of the hemoglobin and is connected into a continuous line. Age, sex, race, annual household income, education level, SBP, DBP, BMI, red blood cell count, albumin, ALP, ALT, blood urea nitrogen, total calcium, iron, CPK, creatinine, glucose, LDH, phosphorus, total bilirubin, total protein, uric acid, albumin creatinine ratio, blood cadmium, blood lead, phosphorus, potassium, triglyceride, LDL-C, HDL-C, total cholesterol, alcohol status, smoking status and diabetes were adjusted.

**Figure 3:**
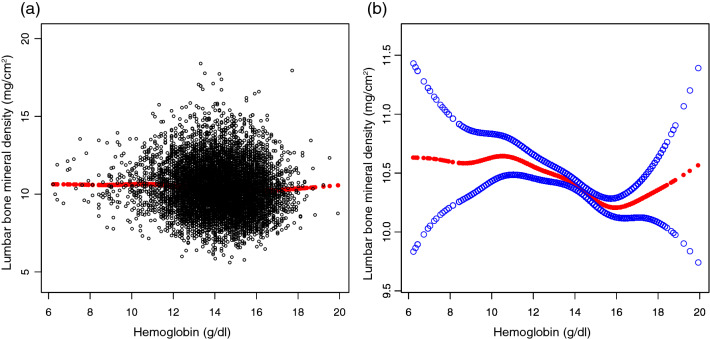
Correlation between hemoglobin and lumbar bone mineral density. (**a**) Each black point represents one sample. (**b**) The area between the two blue dashed lines is represented as a 95% CI. The area between the lines is expressed as 95% CI. Each point represents the size of the hemoglobin and is connected into a continuous line. Age,sex, race, annual household income, education level, SBP, DBP, BMI, red blood cell count, albumin, ALP, ALT, blood urea nitrogen, total calcium, iron, CPK, creatinine, glucose, LDH, phosphorus, total bilirubin, total protein, uric acid, albumin creatinine ratio, blood cadmium, blood lead, phosphorus, potassium, triglyceride, LDL-C, HDL-C, total cholesterol, alcohol status, smoking status and diabetes were adjusted.

After fully adjusting for confounders, HGB was negatively correlated with lumbar spine BMD (β = − 0.0416, P = 0.031873) (Table 2, Fig. 2). However, the negative correlation between HGB and thoracic spine BMD was not statistically significant (β = − 0.0127, P = 0.362458) (Table 2, Fig. 3). In fully adjusted subgroup analyses stratified by sex and race, the negative association between HGB and thoracic spine BMD and lumbar spine BMD was only present in females and other races and races that were not recorded (Tables 3 and 4).

**Table 2:** Relationship between HGB and BMD.

	Non-adjusted	Adjust I	Adjust II
Thoracic spine BMD	0.0716 (0.0551, 0.0881) < 0.000001	− 0.0476 (− 0.0689, − 0.0263) 0.000012	− 0.0127 (− 0.0401, 0.0147) 0.362458
Lumbar spine BMD	− 0.0756 (− 0.0971, − 0.0540) < 0.000001	− 0.0975 (− 0.1259, − 0.0690) < 0.000001	− 0.0416 (− 0.0797, − 0.0036) 0.031873

Expose variable HGB (g/dL), ending variable thoracic Spine BMD and lumbar spine BMD (mg/cm^2^), the results in the table are expressed as β (95% CI).

Model 1, no covariates were adjusted.

Model 2, age, sex, race were adjusted.

Model 3, age, sex, race, annual household income, education level, SBP, DBP, BMI, red blood cell count, albumin, ALP, ALT, blood urea nitrogen, total calcium, iron, CPK, creatinine, glucose, LDH, phosphorus, total bilirubin, total protein, uric acid, albumin creatinine ratio, blood cadmium, blood lead, phosphorus, potassium, triglyceride, LDL-C, HDL-C, total cholesterol, alcohol status, smoking status and diabetes were adjusted.

**Table 3:** Association between HGB and BMD stratified by gender.

	Non-adjusted	Adjust I	Adjust II
**Thoracic spine BMD**			
Male	− 0.0307 (− 0.0632, 0.0018) 0.064372	− 0.0002 (− 0.0323, 0.0319) 0.990719	0.0232 (− 0.0198, 0.0663) 0.290500
Female	− 0.0806 (− 0.1085, − 0.0527) < 0.000001	− 0.0722 (− 0.1003, − 0.0441) < 0.000001	− 0.0438 (− 0.0790, − 0.0086) 0.014779
**Lumbar spine BMD**			
Male	− 0.0927 (− 0.1363, − 0.0491) 0.000031	− 0.0923 (− 0.1362, − 0.0483) 0.000039	− 0.0184 (− 0.0798, 0.0430) 0.556416
Female	− 0.1081 (− 0.1448, − 0.0714) < 0.000001	− 0.0941 (− 0.1308, − 0.0573) < 0.000001	− 0.0780 (− 0.1261, − 0.0299) 0.001499

Expose variable HGB (g/dL), ending variable lumbar spine BMD, the results in the table are expressed as β (95% CI).

Model 1, no covariates were adjusted.

Model 2, age, race were adjusted.

Model 3, age, race, annual household income, education level, SBP, DBP, BMI, red blood cell count, albumin, ALP, ALT, blood urea nitrogen, total calcium, iron, CPK, creatinine, glucose, LDH, phosphorus, total bilirubin, total protein, uric acid, albumin creatinine ratio, blood cadmium, blood lead, phosphorus, potassium, triglyceride, LDL-C, HDL-C, total cholesterol, alcohol status, smoking status and diabetes were adjusted.

In the subgroup analysis stratified by gender, the model is not adjusted for the stratification variable itself.

**Table 4:** Association between HGB and BMD stratified by race.

	Non-adjusted	Adjust I	Adjust II
**Thoracic spine BMD**			
Mexican American	0.1043 (0.0688, 0.1399) < 0.000001	− 0.0082 (− 0.0539, 0.0375) 0.726446	− 0.0246 (− 0.0863, 0.0372) 0.435539
Other Hispanic	0.0837 (0.0300, 0.1375) 0.002332	− 0.0778 (− 0.1486, − 0.0071) 0.031307	− 0.0230 (− 0.1141, 0.0681) 0.620970
Non-Hispanic White	0.0795 (0.0505, 0.1084) < 0.000001	− 0.0402 (− 0.0776, − 0.0027) 0.035695	− 0.0031 (− 0.0522, 0.0460) 0.900976
Non-Hispanic Black	0.0731 (0.0367, 0.1094) 0.000086	− 0.0742 (− 0.1207, − 0.0277) 0.001809	0.0008 (− 0.0582, 0.0599) 0.977939
Non-Hispanic Asian	0.0444 (− 0.0020, 0.0907) 0.061182	− 0.0398 (− 0.1021, 0.0226) 0.211246	0.0075 (− 0.0741, 0.0890) 0.857496
Other Races and Not recorded	0.0271 (− 0.0423, 0.0966) 0.444195	− 0.1110 (− 0.1993, − 0.0227) 0.014051	− 0.1191 (− 0.2329, − 0.0053) 0.040823
**Lumbar spine BMD**			
Mexican American	− 0.0250 (− 0.0761, 0.0262) 0.338566	− 0.0357 (− 0.1024, 0.0311) 0.295251	− 0.0798 (− 0.1715, 0.0118) 0.087958
Other Hispanic	− 0.1062 (− 0.1761, − 0.0363) 0.002979	− 0.1494 (− 0.2436, − 0.0552) 0.001933	− 0.0949 (− 0.2194, 0.0295) 0.135367
Non-Hispanic White	− 0.0657 (− 0.1030, − 0.0284) 0.000566	− 0.0827 (− 0.1317, − 0.0336) 0.000966	− 0.0144 (− 0.0814, 0.0525) 0.672600
Non-Hispanic Black	− 0.1078 (− 0.1548, − 0.0608) 0.000007	− 0.1734 (− 0.2353, − 0.1114) < 0.000001	− 0.0388 (− 0.1208, 0.0433) 0.354759
Non-Hispanic Asian	− 0.0518 (− 0.1124, 0.0088) 0.094161	− 0.0473 (− 0.1300, 0.0354) 0.262388	− 0.0241 (− 0.1353, 0.0870) 0.670396
Other races and not recorded	− 0.1206 (− 0.2065, − 0.0348) 0.006117	− 0.1360 (− 0.2481, − 0.0240) 0.017681	− 0.1617 (− 0.3165, − 0.0069) 0.041218

Expose variable HGB (g/dL), ending variable lumbar spine BMD, the results in the table are expressed as β (95% CI).

Model 1, no covariates were adjusted.

Model 2, age, sex were adjusted.

Model 3, age, sex, annual household income, education level, SBP, DBP, BMI, red blood cell count, albumin, ALP, ALT, blood urea nitrogen, total calcium, iron, CPK, creatinine, glucose, LDH, phosphorus, total bilirubin, total protein, uric acid, albumin creatinine ratio, blood cadmium, blood lead, phosphorus, potassium, triglyceride, LDL-C, HDL-C, total cholesterol, alcohol status, smoking status and diabetes were adjusted.

In the subgroup analysis stratified by race, the model is not adjusted for the stratification variable itself.

**Figure 4:**
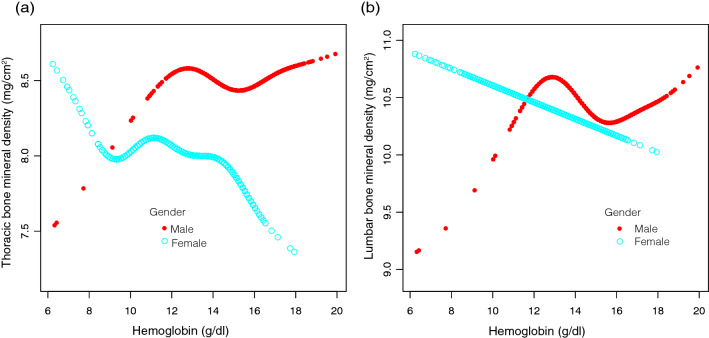
The hemoglobin and bone mineral density relationship, stratified by gender. Age, race, annual household income, education level, SBP, DBP, BMI, red blood cell count, albumin, ALP, ALT, blood urea nitrogen, total calcium, iron, CPK, creatinine, glucose, LDH, phosphorus, total bilirubin, total protein, uric acid, albumin creatinine ratio, blood cadmium, blood lead, phosphorus, potassium, triglyceride, LDL-C, HDL-C, total cholesterol, alcohol status, smoking status and diabetes were adjusted. (**a**) Thoracic BMD; (**b**) Lumbar BMD. Red line: Male; Blue line: Female.

**Figure 5:**
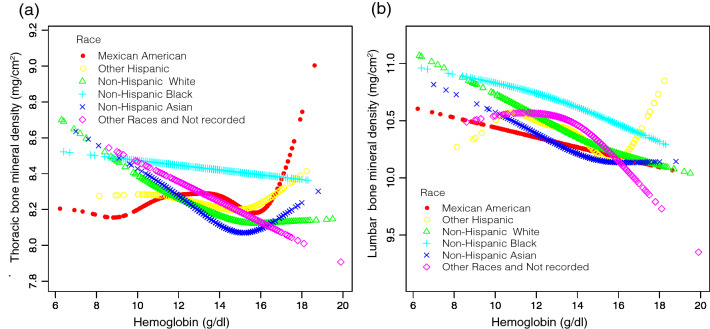
The hemoglobin and bone mineral density relationship, stratified by race. Age, sex, annual household income, education level, SBP, DBP, BMI, red blood cell count, albumin, ALP, ALT, blood urea nitrogen, total calcium, iron, CPK, creatinine, glucose, LDH, phosphorus, total bilirubin, total protein, uric acid, albumin creatinine ratio, blood cadmium, blood lead, phosphorus, potassium, triglyceride, LDL-C, HDL-C, total cholesterol, alcohol status, smoking status and diabetes were adjusted. (**a**) Thoracic BMD; (**b**) Lumbar BMD. Red line: Mexican American; Yellow line: Other Hispanic; Green line: Non-Hispanic White; Light blue line: Non-Hispanic Black; Dark blue line: Non-Hispanic Asian; Purple line: Other Races and Not recorded.

In addition, fully adjusted smoothened plots showed a non-linear relationship between HGB and BMD in the lumbar and thoracic spine after stratification by sex or race (Figs. 4 and 5). Among participants of Non-Hispanic Asian, BMD in the thoracic spine decreased with HGB until the turning point (turning point: HGB 14.9 g/dL). Similarly, there was a turning point between HGB and BMD in the lumbar spine (turning point: HGB 15.6 g/dL). There is also a turning point in other races and races that were not recorded (turning point: HGB 13.4 g/dL) (Table 5).

**Table 5:** Threshold effect analysis of HGB on Thoracic Spine BMD and Lumbar Spine BMD using two-piecewise linear regression.

	Hemoglobin (g/dL)	Adjusted β (95% CI), *p*-value
**Non-Hispanic Asian**		
Thoracic spine BMD	< 14.9	− 0.1 (− 0.2, 0.0) 0.095
	> 14.9	0.2 (0.1, 0.4) 0.002
Lumbar spine BMD	< 15.6	− 0.1 (− 0.2, 0.0) 0.138
	> 15.6	0.4 (0.1, 0.7) 0.012
**Non**− **Hispanic White**		
Thoracic spine BMD	< 15.1	− 0.0 (− 0.1, 0.0) 0.395
	> 15.1	0.1 (− 0.0, 0.2) 0.196
**Other race and not recorded**		
Lumbar spine BMD	< 13.4	0.1 (− 0.1, 0.3) 0.376
	> 13.4	− 0.4 (− 0.6, − 0.2) < 0.001

Age, sex, annual household income, education level, SBP, DBP, BMI, red blood cell count, albumin, ALP, ALT, blood urea nitrogen, total calcium, iron, CPK, creatinine, glucose, LDH, phosphorus, total bilirubin, total protein, uric acid, albumin creatinine ratio, blood cadmium, blood lead, phosphorus, potassium, triglyceride, LDL-C, HDL-C, total cholesterol, alcohol status, smoking status and diabetes were adjusted.

Overall, the relationship between HGB and BMD in the lumbar spine and BMD in the thoracic spine in Non-Hispanic Asian showed a U-shaped curve. In other races and races that were not recorded, there was an inverted U-shaped curve between HGB and lumbar BMD.

## Discussion

The main goal of this study was to clarify whether HGB is correlated with BMD in the lumbar and thoracic spine. In our comprehensive adjusted model multiple linear regression analysis, we found that HGB was negatively correlated with lumbar BMD, and the completely adjusted smoothed curve fits showed a non-linear correlation. When stratified by sex and race, the relationship between HGB and thoracic spine BMD and lumbar spine BMD was only found in women and other races and races that were not recorded. In Non-Hispanic Asian subjects, the relationship between HGB and BMD in the lumbar spine and thoracic spine showed a U-shaped curve. In addition, the relationship between HGB and BMD in the lumbar spine formed an inverted U-shaped curve among participants in other races and those whose race was not reported.

Similarly, a cross-sectional study involving 3626 Korean participants found that HGB levels were inversely associated with low BMD in the lumbar spine among non-anemic adults^30^. Furthermore, in a recently published study, BMD was negatively correlated with HGB levels in younger and older women^31^. imilar results were obtained by Cesari et al. in a survey of 950 older adults^32^. Nevertheless, a study examining the association between serum HGB levels, BMD, and fracture risk using estimated scores from the Fracture Risk Assessment Tool (FRAX) in 662 male patients concluded that HGB was positively associated with BMD but negatively associated with the risk of hip fracture and major osteoporotic fracture^33^. It is possible that age and ethnic differences in the study population, or the limited sample size, may have influenced the results. Based on this, the cross-sectional study has a broad and large sample size, targeting both males and females aged 18 years and older, and provides subgroup analysis across gender and race.

The mechanism of the link between HGB and BMD is not yet clear; however, based on the theory that both osteoblasts and cells of the hematopoietic microenvironment that are responsible for maintaining hematopoietic tissue have a common progenitor, namely mesenchymal stem cells (MSC)^34^, Gurevitch et al. proposed a hypothesis that the differentiation pathways of osteogenesis and the hematopoietic microenvironment compete with each other, with osteogenic stimulation predominating during the growth phase of the organism. Nonetheless, after maturation, there is a gradual increase in differentiation of MSCs towards cells of the hematopoietic microenvironment and a decrease in intraosseous differentiation. This subsequently leads to a reduction in bone mass and enlargement of the bone marrow cavity in hematopoietically active cancellous bones^35^. They speculated that continuous overproduction of blood cells leads to excessive depletion of the hematopoietic system and is a non-negligible component in the etiology of osteoporosis. Blood loss promotes the proliferation of hematopoietic progenitor cells, leading to an increase in the number of hematopoietic cells including osteoblasts, which enhances bone tissue resorption. In addition, a reduction in blood volume stimulates bone development and increases the number of osteoblasts, thereby promoting new bone formation^36^. It has also been shown that acute bleeding stimulates the secretion of bone morphogenetic protein 2 and BMP6 by hematopoietic stem cells, thereby driving MSC differentiation along the osteogenic pathway^37^.

This cross-sectional study not only confirmed the association between HGB and BMD, but also provided substantial clinical significance. The U-shaped curve in the relationship between HGB and BMD in the Non-Hispanic Asian population implies that BMD in the thoracic and lumbar spine may be quietly decreasing as HGB levels approach 14.9 (g/dL) or 15.6 (g/dL) levels. More notably, however, the inverted U-shaped curve in the relationship between HGB and BMD of the lumbar and thoracic spine in people of other races and unreported races demonstrates that for these patients, clinicians should be aware of low levels of HGB while being alert to the risk of reduced bone mass and the need for close monitoring of BMD and early intervention.

This study analyzed NHANES 2013–2018 data conducted by the U.S. Centers for Disease Control and Prevention. Owing to the rigorous design of the NHANES database, the accuracy of the data requires the completion of a large sample size and stratified analysis for this study. It is regrettable that for some confounding factors that may affect the results, such as chronic kidney disease, chronic inflammatory disease, long-term infection, and the use of certain drugs, due to the lack of relevant information in the 2013–2018 NHANES database, this study cannot describe the current cases in the study. Nonetheless, this study relied on a cross-sectional design; therefore, it was not possible to ascertain a causal relationship between BMD and HGB.

## Conclusions

Our study shows that HGB has a non-linear relationship with lumbar and thoracic BMD. Further studies are required to elucidate the mechanisms underlying this association.

## Supplementary Information

Supplementary Information.

## Data Availability

The datasets obtained and analysed during the current study are available in the NHANES [https://www.cdc.gov/nchs/nhanes/index.htm].

## Abbreviations

HGB: Hemoglobin
BMD: Bone mineral density
NHANES: National Health and Nutrition Survey
SBP: Systolic blood pressure
DBP: Diastolic blood pressure
BMI: Body mass index
ALP: Alkaline phosphatase
ALT: Alanine aminotransferase
CPK: Creatine phosphor kinase
TG: Triglyceride
LDH: Lactate dehydrogenase
LDL-C: Low-density lipoprotein cholesterol
HDL-C: High-density lipoprotein cholesterol
RA: Rheumatoid arthritis
COPD: Chronic obstructive pulmonary disease
MECs: Mobile examination centers
CBC: Complete blood count
DXA: Duel energy X-ray absorptiometry
CAPI: Computer-assisted personal interview
STROBE: Strengthening the Reporting of Observational Studies in Epidemiology
FRAX: Fracture Risk Assessment Tool
MSC: Mesenchymal stem cell
