# Supplementary material for: Association of hemoglobin levels with bone mineral density for adults over 18 years of age: a cross-sectional study
Source: Sci Rep. 2022 Jun 15;12:9975. doi: 10.1038/s41598-022-13973-w (PMC9200788; doi:10.1038/s41598-022-13973-w)
Supplement: Supplementary file 1 — Supplementary Information. [file 41598_2022_13973_MOESM1_ESM.docx]

S1: The description of methods, intra-assay and inter-assay CV of the biochemiscal tests

| Standard Biochemistry Profile | Description of Laboratory Methodology | intra-assay and inter-assay CV |
| --- | --- | --- |
|  |  |  |
| Alanine Aminotransferase (ALT) | Aspartate aminotransferase (AST) activity is determined by a modification of the method recommended by the International Federation of Clinical Chemistry (IFCC). AST catalyzes the reaction of alpha-ketoglutarate with L-aspartate to form L-glutamate and oxaloacetate. Under the action of malate dehydrogenase (MDH), oxaloacetate converts to malate, and NADH is oxidized to NAD. The decrease in absorbance of NADH, measured at 340 nm (secondary wavelength = 700 nm), is directly proportional to the serum activity of AST. It is a kinetic rate reaction. | Intra-assay %CV (10 within-day replicates at a concentration of 45.4 U/L) 1.1% Intra-assay %CV (10 within-day replicates at a concentration of 111.5 U/L) 0.5% Inter-assay %CV (between day replicates at a concentration of 21 U/L) 2.4% Inter-assay %CV (between day replicates at a concentration of 133 U/L) 2.0% |
| Total Calcium | The method used to measure total calcium reacts with 5-nitro-5’methyl-BAPTA (NM-BAPTA) under alkaline conditions to form a complex. This complex then reacts with EDTA to form a colored product whose intensity is directly proportional to the concentration of calcium in the specimen. It is measured photometrically at 340 nm. | Intra-assay %CV (10 within-day replicates at a concentration of 8.7 mg/dL) 0.8%  Intra-assay %CV (10 within-day replicates at a concentration of 13.25 mg/dL) 0.5%  Inter-assay %CV (between day replicates at a concentration of 8.56 mg/dL) 1.3%  Inter-assay %CV (between day replicates at a concentration of 14.15 mg/dL) 1.3% |
| Total Bilirubin | The method to measure total bilirubin is coupled with 3,5-dichlorophenyl diazonium in the presence of a solubilizing agent in a strongly acidic medium. The intensity of the red azo dye formed is directly proportional to the total bilirubin and can be determined photometrically (546 nm). | Intra-assay %CV (10 within-day replicates at a concentration of 0.85 mg/dL) 2.1%  Intra-assay %CV (10 within-day replicates at a concentration of 3.78 mg/dL) 0.7%  Inter-assay %CV (between day replicates at a concentration of 0.26 mg/dL) 7.3%  Inter-assay %CV (between day replicates at a concentration of 3.66 mg/dL) 3.3% |
| Total Protein | The total protein method utilizes the biuret reaction with measurement of the final product at 546 nm. Divalent copper reacts in alkaline solution with protein peptide bonds to form the characteristic purple-colored biuret complex. Sodium potassium tartrate prevents the precipitation of copper hydroxide and potassium iodide prevents auto-reduction of copper. The color intensity is directly proportional to the protein concentration. | Intra-assay %CV (10 within-day replicates at a concentration of 4.33 g/dL) 1.1%  Intra-assay %CV (10 within-day replicates at a concentration of 6.13 g/dL) 0.8%  Inter-assay %CV (between day replicates at a concentration of 4.34 g/dL) 2.1%  Inter-assay %CV (between day replicates at a concentration of 6.30 g/dL) 1.6% |
| Phosphorous | The method used to measure phosphorus utilizes ammonium molybdate as the color-forming reagent. Measurement of the final product occurs at 340 nm (secondary wavelength 700 nm). Inorganic phosphate forms an ammonium phosphomolybdate complex having the formula (NH4)3[PO4(MoO3)12] with ammonium molybdate in the presence of sulfuric acid. The concentration of phosphomolybdate formed is directly proportional to the inorganic phosphate concentration. | Intra-assay %CV (10 within-day replicates at a concentration of 4.0 mg/dL) 0.0%  Intra-assay %CV (10 within-day replicates at a concentration of 6.14 mg/dL) 0.8%  Inter-assay %CV (between day replicates at a concentration of 3.66 mg/dL) 1.6%  Inter-assay %CV (between day replicates at a concentration of 6.74 mg/dL) 1.3% |
| Total Cholesterol | The method used to measure cholesterol is an enzymatic method where esterified cholesterol is converted to cholesterol by cholesterol esterase. The resulting cholesterol is then acted upon by cholesterol oxidase to produce cholest-4-en-3-one and hydrogen peroxide. The hydrogen peroxide then reacts with 4-aminophenazone in the presence of peroxidase to produce a colored product that is measured at 505 nm (secondary wavelength = 700 nm). The final step is known as the Trinder reaction. This method is a single reagent, endpoint reaction that is specific for cholesterol. | Intra-assay %CV (10 within-day replicates at a concentration of 90.4 mg/dL) 0.8%  Intra-assay %CV (10 within-day replicates at a concentration of 174 mg/dL) 0.9%  Inter-assay %CV (between day replicates at a concentration of 179 mg/dL) 2.1%  Inter-assay %CV (between day replicates at a concentration of 259 mg/dL) 2.2% |
| Lactate Dehydrogenase LDH | The Roche method of LDH measurement is derived from the formulation recommended by the International Federation of Clinical Chemistry (IFCC) and is optimized for performance and stability. In the presence of cofactor NAD+, LDH converts L-lactate to pyruvate. NAD+ is reduced to NADH during this reaction. The initial rate of NADH formation is directly proportional to the catalytic LDH activity and is determined by measuring the increase in absorbance at 340 nm. This is a kinetic (Rate-A) reaction. | Intra-assay %CV (10 within-day replicates at a concentration of 189.0 U/L) 0.6%  Intra-assay %CV (10 within-day replicates at a concentration of 305.9 U/L) 0.5%  Inter-assay %CV (between day replicates at a concentration of 161.1 U/L) 2.2%  Inter-assay %CV (between day replicates at a concentration of 305.5 U/L) 2.6% |
| Iron | The Roche method of iron measurement is a three-step process using FerroZine reagent: Fe3+ is liberated from transferrin by acid/detergent, Fe3+ is reduced to Fe2+ by ascorbate, and the reduced iron then reacts with the FerroZine reagent to form a colored complex. The intensity of this final product is directly proportional to the iron concentration in the specimen. | Intra-assay %CV (10 within-day replicates at a concentration of 108 ug/dL) 0.8%  Intra-assay %CV (10 within-day replicates at a concentration of 247 ug/dL) 0.3%  Inter-assay %CV (between day replicates at a concentration of 77 ug/dL) 2.7%  Inter-assay %CV (between day replicates at a concentration of 251 ug/dL) 1.7% |
| Creatinine Phosphokinase (CPK) | The method to measure creatine phosphokinase (CPK) or creatine kinase (CK) utilizes a coupled enzyme reaction. Creatine phosphate and adenosine diphosphate (ADP) are acted upon by CK in the serum specimen. Creatine and ATP are produced from this reaction, and ATP reacts with glucose under the action of hexokinase to produce glucose-6-phosphate and ADP. The glucose-6-phosphate reacts with NADP under the action of glucose-6-phosphate dehydrogenase to produce NADPH and a by-product. The photometrically (340 nm) measured rate of NADPH formation is directly proportional to the CK activity in the specimen. | Intra-assay %CV (10 within-day replicates at a concentration of 163 U/L) 1.0%  Intra-assay %CV (10 within-day replicates at a concentration of 462 U/L) 1.7%  Inter-assay %CV (between day replicates at a concentration of 155 U/L) 1.3%  Inter-assay %CV (between day replicates at a concentration of 485 U/L) 1.7% |
| Glucose | The method to measure glucose utilizes an enzymatic method that converts glucose to glucose-6-phosphate (G-6-P) by hexokinase in the presence of ATP, a phosphate donor. Glucose-6-phosphate dehydrogenase then converts the G-6-P to gluconate-6-P in the presence of NADP+. As the NADP+ is reduced to NADPH during this reaction, the resulting increase in absorbance at 340 nm (secondary wavelength = 700 nm) is measured. This is an endpoint reaction that is specific for glucose. | Intra-assay %CV (10 within-day replicates at a concentration of 86.7mg/dL) 0.8% Intra-assay %CV (10 within-day replicates at a concentration of 222.4 mg/dL) 0.8% Inter-assay %CV (between day replicates at a concentration of 96.9 mg/dL) 0.9% Inter-assay %CV (between day replicates at a concentration of 221.4 mg/dL) 1.1% |
| Creatinine | Creatinine is measured using an enzymatic method in which creatinine is converted to creatine under the activity of creatininase. Creatine is then acted upon by creatinase to form sarcosine and urea. Sarcosine oxidase converts sarcosine to glycine and hydrogen peroxide, and the hydrogen peroxide reacts with a chromophore in the presence of peroxidase to produce a colored product that is measured at 546 nm (secondary wavelength = 700 nm). This is an endpoint reaction that agrees well with recognized HPLC methods, and it has the advantage over Jaffe picric acid-based methods that are susceptible to interferences from non-creatinine chromogens. | Intra-assay %CV (10 within-day replicates at a concentration of 1.00 mg/dL) 1.0%  Intra-assay %CV (10 within-day replicates at a concentration of 3.53 mg/dL) 0.9%  Inter-assay %CV (between day replicates at a concentration of 0.83 mg/dL) 3.0%  Inter-assay %CV (between day replicates at a concentration of 3.85 mg/dL) 1.9% |
| Album | The method to measure albumin concentration utilizes the dye bromcresol purple (BCP). When the dye binds selectively with albumin in a pH range of 5.2-6.8, a color change occurs that is measured at 600 nm.  The secondary wavelength is 700 nm. This is a 2-point, endpoint reaction that is specific for albumin. | Intra-assay %CV (10 within-day replicates at a concentration of 3.0 g/dL) 0.0%  Intra-assay %CV (10 within-day replicates at a concentration of 4.6 g/dL) 0.7%  Inter-assay %CV (between day replicates at a concentration of 3.90 g/dL) 2.6%  Inter-assay %CV (between day replicates at a concentration of 2.68 g/dL) 2.2% |
| Alkaline Phosphatase (ALP) | The method to measure alkaline phosphatase (ALP) utilizes a simple reaction wherein ALP acts upon a substrate (p-nitrophenol phosphate, or PNPP) in the presence of magnesium and zinc activators to form a colored product (p-nitrophenol) whose appearance is measured at 450 nm. The rate of p-nitrophenol formation is | Inter-assay %CV (between day replicates at a concentration of 64 U/L) 1.7%  Inter-assay %CV (between day replicates at a concentration of 215 U/L) 3.3% |
| Blood Urea Nitrogen | The method to measure blood urea nitrogen utilizes a coupled enzyme reaction (urease, followed by glutamate dehydrogenase), with measurement of NADH (converting to NAD+) occurring at 340 nm. | Intra-assay %CV (10 within-day replicates at a concentration of 18.6 mg/dL) 1.3% Intra-assay %CV (10 within-day replicates at a concentration of 57.19 mg/dL) 1.3% Inter-assay %CV (between day replicates at a concentration of 15.3 mg/dL) 2.0% Inter-assay %CV (between day replicates at a concentration of 54.0 mg/dL) 2.0% |
| Uric Acid | In this method uric acid is oxidized by uricase. Then the peroxide produced from this reaction is acted upon by peroxidase in the presence of 4 aminophenazone to produce a measurable colored product. It is a two-point, endpoint reaction, with measurement occurring at 546 nm (secondary wavelength 700 nm). | Intra-assay %CV (10 within-day replicates at a concentration of 4.99 mg/dL) 0.6%  Intra-assay %CV (10 within-day replicates at a concentration of 6.94 mg/dL) 1.0%  Inter-assay %CV (between day replicates at a concentration of 5.51 mg/dL) 0.7%  Inter-assay %CV (between day replicates at a concentration of 9.59 mg/dL) 1.6% |
| Triglycerides | The method used to measure triglyceride is based on the work by Wahlefeld using a lipoprotein lipase from microorganisms for the rapid and complete hydrolysis of triglycerides to glycerol followed by oxidation to dihydroxyacetone phosphate and hydrogen peroxide. The hydrogen peroxide produced then reacts with 4-aminophenazone and 4-chlorophenol under the catalytic action of peroxidase to form a red dyestuff (Trinder endpoint reaction). The color intensity of the red dyestuff formed is directly proportional to the triglyceride concentration and can be measured photometrically. | Intra-assay %CV (10 within-day replicates at a concentration of 65.8 mg/dL) 0.6% Intra-assay %CV (10 within-day replicates at a concentration of 361.5 mg/dL) 0.5% Inter-assay %CV (between day replicates at a concentration of 112.0 mg/dL) 1.3% Inter-assay %CV (between day replicates at a concentration of 196.5 mg/dL) 2.0% |
| Potassium | Potassiumion concentration is measured by electrolyte activity in solution. This method utilizes an indirect (specimen is diluted by the instrument prior to analysis) ion-selective electrode (ISE) method for determination of the serum electrolyte concentrations. | Intra-assay %CV (10 within-day replicates at a concentration of 3.16 mmol/L) 0.0%  Intra-assay %CV (10 within-day replicates at a concentration of 5.96 mmol/L) 0.1%  Inter-assay %CV (between day replicates at a concentration of 3.19 mmol/L) 0.7%  Inter-assay %CV (between day replicates at a concentration of 4.54 mmol/L) 0.9% |

The contents of the table are derived from https://wwwn.cdc.gov
